# Supplementary material for: Different patterns of structural network impairments in two amyotrophic lateral sclerosis subtypes driven by 18F-fluorodeoxyglucose positron emission tomography/magnetic resonance hybrid imaging
Source: Brain Commun. 2024 Jul 9;6(5):fcae222. doi: 10.1093/braincomms/fcae222 (PMC11368155; doi:10.1093/braincomms/fcae222)
Supplement: fcae222_Supplementary_Data [file fcae222_supplementary_data.docx]

**Supplementary Material**

**Supplementary Figure 1.** **Distribution of labels with decreases in dNE and changes in SUVR based on the AAL**


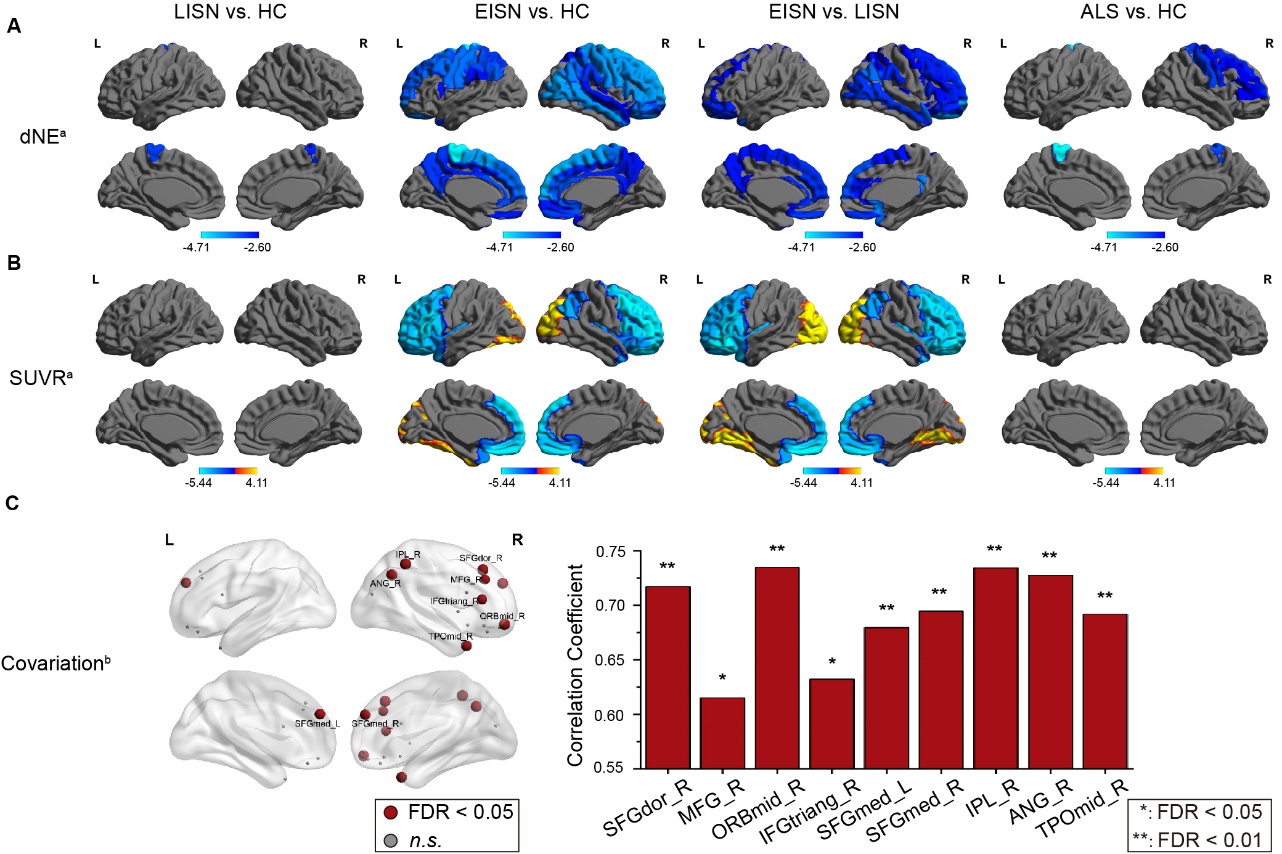
A, Distribution pattern of labels with decreases in dNE. Color represent *P* values of the labels. B, Distribution pattern of labels with decreases and increases in SUVR. Color represent *P* values of the labels. C, Labels with related decreases in dNE and SUVR in the EISN subgroup. The volumes of the red balls represent the Spearman r values of the labels. ALS indicates amyotrophic lateral sclerosis; ANG, Angular gyrus; dNE, nodal efficiency of the DTI-based brain network; EISN, extensively impaired structural network; HC, healthy control; IFGtriang, Inferior frontal gyrus, triangular part; IPL, Inferior parietal, but supramarginal and angular gyri; LISN, locally impaired structural network; MFG, Middle frontal gyrus; ORBmid, Middle frontal gyrus, orbital part; SFGdor, Superior frontal gyrus, dorsolateral; SFGmed, Superior frontal gyrus, medial; SUVR, standardized uptake value ratio; TPOmid, Temporal pole: middle temporal gyrus.

^a^*P* < 0.05, FDR correction.

^b^Labels with related decreases in dNE and SUVR in the EISN subgroup.

**Supplementary Figure 2.** **The impaired structural subnetworks in group comparisons based on the AAL**


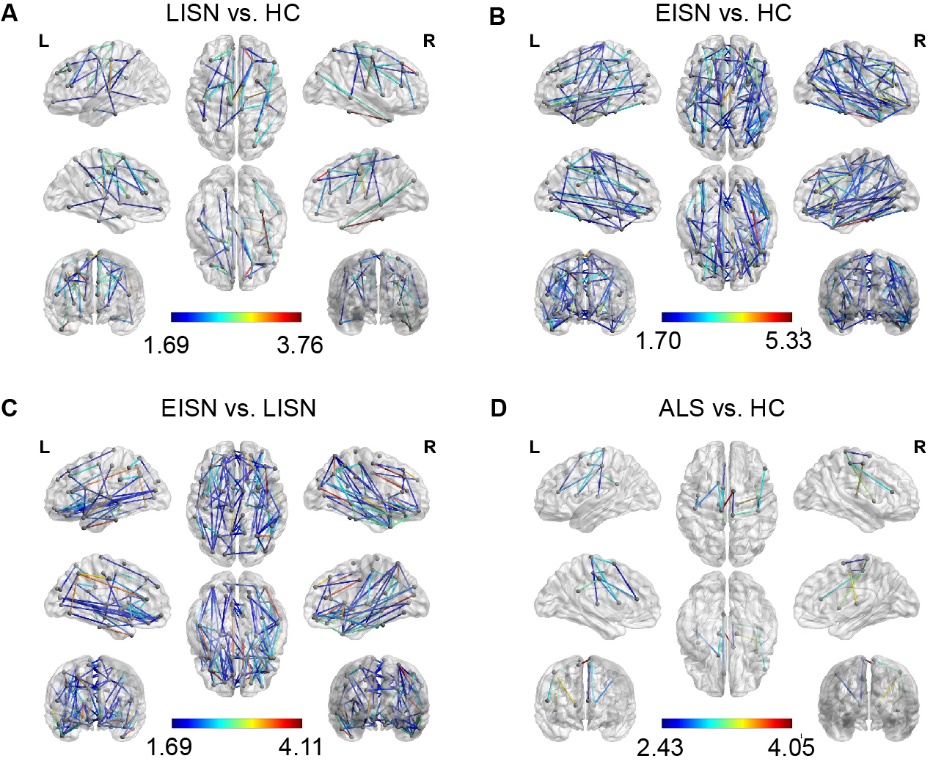


The impaired structural subnetworks in LISN vs. HC (A), EISN vs. HC (B), EISN vs. LISN (C), and ALS vs. HC (D) comparisons (*P* < 0.05, permutation test, Bonferroni correction) by network-based statistics (NBS). Color represent *P* values of the edges constituting the impaired subnetworks. ALS indicates amyotrophic lateral sclerosis; EISN, extensively impaired structural network; HC, healthy control; LISN, locally impaired structural network.

**Supplementary Table 1. Missense mutations in seven patients with ALS**

| **Patient** | **Gene** | **Nucleotide change** | **Amino acid change** | **Pathogenicity** | **Reference** |
| --- | --- | --- | --- | --- | --- |
| FALS | *SOD1* | c.43G>A | p.V15M | pathogenic | Deng et al., 1995^1^ |
| SALS | *SOD1* | c.125G>A | p.G42D | pathogenic | Rosen et al., 1993^2^ |
| SALS | *TARDBP* | c.881G>T | p.G294V | likely pathogenic | Corrado et al., 2009^3^ |
| SALS | *TARDBP* | c.1147A>G | p.I383V | likely pathogenic | Rutherford et al., 2008^4^ |
| SALS | *NEFH* | c.806C>A | p.S269X | likely pathogenic | unreported |
| SALS | *TRPM7* | c.2525C>T | p.T842M | uncertain | Xu et al, 2018^5^ |
| SALS | *SQSTM1* | c.1208C>G | p.S403C | uncertain | unreported |

ALS, amyotrophic lateral sclerosis; FALS, familial ALS; SALS, sporadic ALS.

**Supplementary Table 2. Comparisons of decreases in dNE between groups with the BNA**

|  | **Label ID** | **Gyrus^a^** | **Modified cyto-architectonic** | ***P*^b^** | ***t*** |
| --- | --- | --- | --- | --- | --- |
| LISN vs. HC | | | | | |
| Label 1 | 60 | PrG_R | A4t, area 4(trunk region) | 0.00 | -3.94 |
| Label 2 | 59 | PrG_L | A4t, area 4(trunk region) | 0.01 | -3.53 |
| Label 3 | 57 | PrG_L | A4ul, area 4(upper limb region) | 0.01 | -3.43 |
| Label 4 | 58 | PrG_R | A4ul, area 4(upper limb region) | 0.02 | -3.13 |
| Label 5 | 67 | PCL_L | A4ll, area 4, (lower limb region) | 0.02 | -3.01 |
| EISN vs. HC | | | | | |
| Label 1 | 59 | PrG_L | A4t, area 4(trunk region) | 0.00 | -4.71 |
| Label 2 | 162 | PoG_R | A1/2/3tru, area 1/2/3(trunk region) | 0.00 | -4.56 |
| Label 3 | 130 | SPL_R | A5l, lateral area 5 | 0.00 | -4.46 |
| Label 4 | 118 | PhG_R | TI, area TI(temporal agranular insular cortex) | 0.00 | -4.44 |
| Label 5 | 132 | SPL_R | A7pc, postcentral area 7 | 0.00 | -4.40 |
| EISN vs. LISN | | | | | |
| Label 1 | 122 | pSTS_R | rpSTS, rostroposterior superior temporal sulcus | 0.00 | -4.01 |
| Label 2 | 118 | PhG_R | TI, area TI(temporal agranular insular cortex) | 0.01 | -3.84 |
| Label 3 | 70 | STG_R | A38m, medial area 38 | 0.01 | -3.41 |
| Label 4 | 177 | CG_L | A24rv, rostroventral area 24 | 0.01 | -3.31 |
| Label 5 | 86 | MTG_R | A37dl, dorsolateral area 37 | 0.01 | -3.29 |
| ALS vs. HC | | | | | |
| Label 1 | 60 | PrG_R | A4t, area 4(trunk region) | 0.00 | -5.20 |
| Label 2 | 59 | PrG_L | A4t, area 4(trunk region) | 0.00 | -4.84 |
| Label 3 | 57 | PrG_L | A4t, area 4(trunk region) | 0.00 | -4.23 |
| Label 4 | 162 | PoG_R | A1/2/3tru, area 1/2/3(trunk region) | 0.01 | -4.02 |
| Label 5 | 58 | PrG_R | A4ul, area 4(upper limb region) | 0.01 | -3.86 |

ALS, amyotrophic lateral sclerosis; CG, Cingulate gyrus; EISN, extensively impaired structural network; HC, healthy control; L, Left; LISN, locally impaired structural network; MTG, Middle temporal gyrus; PCL, Paracentral lobule; PhG, Parahippocampal gyrus; PoG, Postcentral gyrus; PrG, Precentral gyrus; pSTS, Posterior superior temporal sulcus; R, right; SFG, Superior frontal gyrus; SPL, Superior parietal lobule; STG, Superior temporal gyrus.

^a^Gyrus including label with top five significance.

^b^FDR correction.

**Supplementary Table 3. Comparisons in SUVR between groups with the BNA**

|  | **Label ID** | **Gyrus^a^** | **Modified cyto-architectonic** | ***P*^b^** | ***t*** |
| --- | --- | --- | --- | --- | --- |
| LISN vs. HC^c^ | | | | | |
| Label 1 | 203 | LOcC_L | OPC, occipital polar cortex | 0.03 | -2.89 |
| EISN vs. HC^c^ | | | | | |
| Label 1 | 51 | OrG_L | A12/47l, lateral area 12/47 | 0.00 | -4.80 |
| Label 2 | 48 | OrG_B | A11m, medial area 11 | 0.00 | -4.75 |
| Label 3 | 5 | SFG_L | A9l, lateral area 9 | 0.00 | -4.73 |
| Label 4 | 26 | MFG_R | A6vl, ventrolateral area 6 | 0.00 | -4.73 |
| Label 5 | 24 | MFG_R | A8vl, ventrolateral area 8 | 0.00 | -4.69 |
| EISN vs. LISN^c^ | | | | | |
| Label 1 | 36 | IFG_R | A45r, rostral area 45 | 0.00 | -5.47 |
| Label 2 | 44 | OrG_R | A12/47o, orbital area 12/47 | 0.00 | -5.36 |
| Label 3 | 43 | OrG_L | A12/47o, orbital area 12/47 | 0.00 | -4.90 |
| Label 4 | 81 | MTG_L | A21c, caudal area 21 | 0.00 | -4.73 |
| Label 5 | 41 | OrG_L | A14m, medial area 14 | 0.00 | -4.67 |
| ALS vs. HC^c^ | - | - | - | - | - |
| LISN vs. HC^d^ | | | | |  |
| Label 1 | 131 | SPL_L | A7pc, postcentral area 7 | 0.01 | 3.48 |
| Label 2 | 125 | SPL_L | A7r, rostral area 7 | 0.03 | 2.89 |
| EISN vs. HC^d^ | | | | | |
| Label 1 | 202 | LOcC_R | V5/MT+, area V5/MT+ | 0.00 | 4.03 |
| Label 2 | 233 | Tha_L | mPMtha, pre-motor thalamus | 0.00 | 3.94 |
| Label 3 | 201 | LOcC_L | V5/MT+, area V5/MT+ | 0.00 | 3.72 |
| Label 4 | 131 | SPL_L | A7pc, postcentral area 7 | 0.00 | 3.64 |
| Label 5 | 149 | PCun_L | A5m, medial area 5(PEm) | 0.00 | 3.57 |
| EISN vs. LISN^d^ | | | | | |
| Label 1 | 107 | FuG_L | A37lv, lateroventral area 37 | 0.00 | 4.44 |
| Label 2 | 201 | LOcC_L | V5/MT+, area V5/MT+ | 0.00 | 4.33 |
| Label 3 | 205 | LOcC_L | iOccG, inferior occipital gyrus | 0.00 | 4.29 |
| Label 4 | 202 | LOcC_R | V5/MT+, area V5/MT+ | 0.00 | 4.10 |
| Label 5 | 206 | LOcC_R | iOccG, inferior occipital gyrus | 0.00 | 4.06 |
| ALS vs. HC^d^ | - | - | - | - | - |

ALS, amyotrophic lateral sclerosis; EISN, extensively impaired structural network; FuG, Fusiform gyrus; HC, healthy control; IFG, Inferior frontal gyrus; L, Left; LISN, locally impaired structural network; LOcC, Lateral occipital cortex; MFG, Middle frontal gyrus; MTG, Middle temporal gyrus; OrG, Orbital gyrus; PCun, Precuneus; R, Right; SFG, Superior frontal gyrus; SPL, Superior parietal lobule; Tha, Thalamus.

^a^Gyrus including label with top five significance.

^b^FDR correction.

^c^Comparisons of decreases in SUVR between groups.

^d^Comparisons of increases in SUVR between groups.

**Supplementary Table 4. ^18^F-FDG metabolism by the general observation in ALS patients**

|  | **LISN (*n* = 36)** | | **EISN (*n* = 14)** | |
| --- | --- | --- | --- | --- |
|  | **ALS-cn** | **ALS-plus** | **ALS-cn** | **ALS-plus** |
| normal ^18^F-FDG metabolism | 13 | 9 | 5 | 1 |
| ^18^F-FDG hypometabolism | 9 | 5 | 1 | 7 |

ALS, amyotrophic lateral sclerosis; ALS-cn, amyotrophic lateral sclerosis with normal cognition; ALS-plus, amyotrophic lateral sclerosis with cognitive impairment, behavior impairment, cognitive and behavior impairment, and frontotemporal dementia; EISN, extensively impaired structural network; LISN, locally impaired structural network.

**Supplementary Table 5. Labels with related decreases in dNE and SUVR in the EISN group with the BNA**

| **Lable ID** | **Gyrus** | **Modified cyto-architectonic** | **Spearman *r*** | ***P*^a^** |
| --- | --- | --- | --- | --- |
| 4 | SFG_R | A8dl, dorsolateral area 8 | 0.76 | 0.02 |
| 26 | MFG_R | A6vl, ventrolateral area 6 | 0.73 | 0.02 |
| 34 | IFG_R | A45c, caudal area 45 | 0.72 | 0.03 |
| 40 | IFG_R | A44v, ventral area 44 | 0.78 | 0.02 |
| 84 | MTG_R | A21r, rostral area 21 | 0.82 | 0.01 |
| 138 | IPL-R | A39rd, rostrodorsal area 39 (Hip3) | 0.80 | 0.01 |
| 142 | IPL-R | A40c, caudal area 40 (PFm) | 0.85 | 0.00 |

BNA, Brainnetome Atlas; dNE, nodal efficiency of the DTI-based brain network; EISN, extensively impaired structural network; IFG, inferior frontal gyrus; IPL, inferior parietal lobule; MFG, middle frontal gyrus; MTG, middle temporal gyrus; R, right; SFG, superior frontal gyrus; SUVR, standardized uptake value ratio.

^a^FDR correction.

**Supplementary Table 6. Percentage of labels with decreases in dNE and SUVR in cognition-related networks**

|  | **Group Comparisons** | **VN**  **BNA (AAL)** | **SMN**  **BNA (AAL)** | **DAN**  **BNA (AAL)** | **VAN**  **BNA (AAL)** | **LN**  **BNA (AAL)** | **FPN**  **BNA (AAL)** | **DN**  **BNA (AAL)** |
| --- | --- | --- | --- | --- | --- | --- | --- | --- |
| dNE | LISN vs. HC | 0  (0) | 7.40  (31.47) | 0  (11.68) | 0.58  (5.11) | 0  (0) | 0  (2.19) | 0  (0.74) |
|  | EISN vs. HC | 11.15  (9.74) | 76.97  (58.46) | 72.93  (55.44) | 74.83  (47.46) | 42.52  (49.02) | 80.45  (69.05) | 71.84  (44.12) |
|  | EISN vs. LISN | 9.68  (1.98) | 27.03  (7.27) | 38.95  (15.83) | 44.87  (14.62) | 46.47  (38.47) | 66.86  (24.77) | 54.34  (23.69) |
|  | ALS vs. HC | 0  (0) | 29.05  (41.44) | 11.37  (16.39) | 9.39  (8.87) | 0  (0) | 26.48  (9.29) | 6.68  (3.68) |
| SUVR | LISN vs. HC | 8.08  (0) | 1.93  (0) | 0.59  (0) | 0  (0) | 0  (0) | 0  (0) | 0  (0) |
|  | EISN vs. HC | 22.20  (22.54) | 7.86  (6.09) | 18.04  (18.83) | 38.04  (34.30) | 53.12  (55.26) | 75.49  (65.27) | 55.67  (47.31) |
|  | EISN vs. LISN | 58.51  (52.13) | 8.56  (6.09) | 19.59  (21.79) | 41.90  (34.30) | 57.75  (55.26) | 74.45  (65.43) | 63.09  (47.88) |
|  | ALS vs. HC | 0  (0) | 0  (0) | 0  (0) | 0  (0) | 0  (0) | 0  (0) | 0  (0) |

AAL, automated anatomical labelling; ALS, amyotrophic lateral sclerosis; BNA, Brainnetome Atlas; DAN, dorsal attention network; DN, default network; dNE, nodal efficiency of the DTI-based brain network; EISN, extensively impaired structural network; FPN, frontoparietal network; HC, healthy control; LISN, locally impaired structural network; LN, limbic network; SMN, somatomotor network; SUVR, standardized uptake value ratio; VAN, ventral attention network; VN, visual network.

**Supplementary Table 7.** **Group comparisons of global measures of structural network with the AAL**

|  | **LISN, EISN, vs. HC** | **LISN vs. HC**  ***P*  *t*** | **EISN vs. HC**  ***P* *t*** | **EISN vs. LISN**  ***P* *t*** | **ALS vs. HC**  ***P* *t*** |
| --- | --- | --- | --- | --- | --- |
| NGE | 0.00 (0.01)^a^ | 0.23 1.63 | 0.00^b^ 3.71 | 0.02 b 2.61 | 0.03 (0.19)^c^ -2.26 |
| NLE | 0.03 (0.12)^a^ | 0.26 1.58 | 0.03^b^ 2.58 | 0.32 1.44 | 0.09 (0.30)^c^ -1.75 |

ALS, amyotrophic lateral sclerosis; AAL, automated anatomical labelling; EISN, extensively impaired structural network; HC, healthy control; LISN, locally impaired structural network; NGE, network global efficiency; NLE, network local efficiency.

^a^*P* value of Kruskal–Wallis ANOVA, the FDR correction *P* value in the bracket.

^b^Values of measures in the latter group are higher than that in the former group.

^c^*P* value of Wilcoxon rank-sum test, the FDR correction *P* value in the bracket.

**Supplementary Table 8.** **Labels with related decreases in dNE and SUVR in the EISN subgroup with the AAL**

| **Lable ID** | **Regions** | **Spearman *r*** | ***P*^a^** |
| --- | --- | --- | --- |
| 4 | SFGdor_R, superior frontal gyrus, dorsolateral | 0.72 | 0.00 |
| 8 | MFG_R, middle frontal gyrus | 0.62 | 0.02 |
| 10 | ORBmid_R, middle frontal gyrus, orbital part | 0.73 | 0.00 |
| 14 | IFGtriang_R, inferior frontal gyrus, triangular part | 0.63 | 0.02 |
| 23 | SFGmed_L, superior frontal gyrus, medial | 0.68 | 0.01 |
| 24 | SFGmed_R, superior frontal gyrus, medial | 0.69 | 0.01 |
| 62 | IPL_R, inferior parietal, but supramarginal and angular gyri | 0.73 | 0.00 |
| 66 | ANG_R, angular gyrus | 0.73 | 0.00 |
| 88 | TPOmid_R, temporal pole: middle temporal gyrus | 0.69 | 0.01 |

AAL, automated anatomical labelling; dNE, nodal efficiency of the DTI-based brain network; EISN, extensively impaired structural network; SUVR, standardized uptake value ratio.

^a^FDR correction.

**Supplementary references**

1.Deng HX, Tainer JA, Mitsumoto H, et al. Two novel SOD1 mutations in patients with familial amyotrophic lateral sclerosis. *Hum Mol Genet.* 1995;4(6):1113-1116.

2.Rosen DR Mutations in Cu/Zn superoxide dismutase gene are associated with familial amyotrophic lateral sclerosis. *Nature.* 1993;364(6435):362.

3.Corrado L, Ratti A, Gellera C, et al. High frequency of TARDBP gene mutations in Italian patients with amyotrophic lateral sclerosis. *Hum Mutat.* 2009;30(4):688-694.

4.Rutherford NJ, Zhang YJ, Baker M, et al. Novel mutations in TARDBP (TDP-43) in patients with familial amyotrophic lateral sclerosis. *PLoS Genet.* 2008;4(9):e1000193.

5.Xu Y, Liu X, Shen J, et al. The Whole Exome Sequencing Clarifies the Genotype- Phenotype Correlations in Patients with Early-Onset Dementia. *Aging Dis.* 2018;9(4):696-705.
